# Immunogenicity and protective potential of a *Plasmodium* spp. enolase peptide displayed on archaeal gas vesicle nanoparticles

**DOI:** 10.1186/s12936-015-0914-x

**Published:** 2015-10-14

**Authors:** Sneha Dutta, Priya DasSarma, Shiladitya DasSarma, Gotam K. Jarori

**Affiliations:** Department of Biological Sciences, Tata Institute of Fundamental Research, Homi Bhabha Road, Colaba, Mumbai, 400005 India; Department of Microbiology and Immunology, Institute of Marine and Environmental Technology, University of Maryland School of Medicine, Baltimore, MD 21202 USA

**Keywords:** *Plasmodium falciparum* enolase, Protective epitope, *Halobacterium* gas vesicles, Nanoparticles

## Abstract

**Background:**

*Plasmodium falciparum* enolase has been shown to localize on the surface of merozoites and ookinetes. Immunization of mice with recombinant *Plasmodium* enolase (rPfeno) showed partial protection against malaria. Anti-rPfeno antibodies inhibited growth of the parasite in in vitro cultures and blocked ookinete invasion of mosquito midgut epithelium. It is hypothesized that parasite specific moonlighting functions (e.g. host cell invasion) may map on to unique structural elements of Pfeno. Since enolases are highly conserved between the host and the parasite, a parasite-specific epitope of enolase was displayed on novel protein nanoparticles produced by a halophilic Archaeon *Halobacterium* sp. NRC-1 and tested their ability to protect mice against live challenge.

**Methods:**

By genetic engineering, a *Plasmodium*-enolase specific peptide sequence ^104^EWGWS^108^ with protective antigenic potential was inserted into the *Halobacterium* gas vesicle protein GvpC, a protein localized on the surface of immunogenic gas vesicle nanoparticles (GVNPs). Two groups of mice were immunized with the wild type (WT) and the insert containing recombinant (Rec) GVNPs respectively. A third group of mice was kept as un-immunized control. Antibody titres were measured against three antigens (i.e. WT-GVNPs, Rec-GVNPs and rPfeno) using ELISA. The protective potential was determined by measuring percentage parasitaemia and survival after challenge with the lethal strain *Plasmodium yoelii* 17XL.

**Results:**

Rec-GVNP-immunized mice showed higher antibody titres against rPfeno and Rec-GVNPs, indicating that the immunized mice had produced antibodies against the parasite enolase-specific insert sequence. Challenging the un-immunized, WT-GVNP and Rec-GVNP-immunized mice with a lethal strain of mice malarial parasite showed significantly lower parasitaemia and longer survival in the Rec-GVNP-immunized group as compared to control groups. The extent of survival advantage in the Rec-GVNP-group showed positive correlation with anti-rPfeno antibody titres while the parasitaemia showed a negative correlation. These results indicate that the parasite enolase peptide insert displayed on *Halobacterium* GVNPs is a good candidate as a protective antigenic epitope.

**Conclusion:**

The work reported here showed that the parasite-specific peptide sequence is a protective antigenic epitope. Although antibody response of B-cells to the guest sequence in Rec-GVNPs was mild, significant advantage in the control of parasitaemia and survival was observed. Future efforts are needed to display multiple antigens with protective properties to improve the performance of the GVNP-based approach.

## Background

Malaria is a major health problem in developing countries that caused ~584,000 deaths in 2013 [1]. Multiple drugs are available for the treatment of this disease and several new ones are constantly being introduced. However, the emergence of resistance to drugs is rapidly diminishing the effectiveness of current treatments [2]. As a result, the development of an effective vaccine is highly desirable as a major preventative tool. Thus far, efforts have been partially successful with a candidate vaccine, RTS,S currently in Phase-3 trials and one version (*Mosquirix* produced by GlaxoSmithKline Biologicals) was recently licensed by the European Medicines Agency for use in infants and children [3, 4].

*Plasmodium* has a complex life cycle and invades host cells at three different stages. The sporozoites invade hepatocytes, the merozoites invade RBCs and the ookinetes invade mosquito midgut epithelium [5]. The components of the molecular machinery involved in recognition and invasion serve as attractive vaccine candidates. Several molecules involved in the invasion of RBCs [6], hepatocytes [7, 8] and mosquito midgut [9] have been tested for their protective antigenicity against malaria [3]. However, due to the magnitude of the challenges of the disease, sustained efforts are still needed for the continued identification and validation of additional novel antigens with protective potential against malaria.

Apart from the antigens that express only on the surface of invasive stages, certain housekeeping proteins have also been found to localize onto the cell surface. The glycolytic enzyme enolase is one such protein that is present on both merozoite [10] and ookinete [11] cell surfaces. In many other pathogenic and nonpathogenic cells, enolase acts as a cell surface receptor for plasminogen to assist the cells/organism to establish or move through the extra-cellular matrix [12, 13]. In *Plasmodium* spp. ookinetes, surface enolase bound plasminogen helps in digestion of the peritrophic matrix of the mosquito mid gut epithelium. This function is known to map onto a lysine motif DKSLVK of *Plasmodium falciparum* enolase (Pfeno) [11]. On the ookinete surface, enolase also functions as a ligand that recognizes specific receptors on the mosquito midgut epithelium [14]. Anti-Pfeno antibodies have been shown to block both these functions resulting in the disruption of parasite transmission. In merozoites, its role as a protective antigen became evident from the observations that immunization of mice with rPfeno resulted in partial protection against malaria.

Further evidence for the protective antigenic ability of Pfeno was from the observed inhibition of parasite growth in in vitro cultures by anti-rPfeno antibodies [15]. However, the structural elements of Pfeno involved in the protective antigenic functions are still unknown. Since invasion of host cells is unique to the parasite, it is likely to involve a motif that is structurally dissimilar to host enolases. Pfeno has one such motif that is a sequence of five amino acids present as an insert in a surface loop region of the protein [16]. Interestingly, the possibility of the involvement of this motif in invasion related function was indicated by the observation that Pfeno immunized mice surviving lethal parasite challenge had high titres of antibodies directed against this region [17].

To further test the possibility of the pentapeptide insert being a protective epitope, gas vesicle protein nanoparticles (GVNPs) were employed for antigen display and as a delivery vehicle [18]. Nanoparticle-based vaccines have certain advantages due to reduced risk compared to live vaccines, and enhanced protection. Biologically compatible nanoparticles can simultaneously function as both an adjuvant and a delivery system, by presenting antigens to the antigen presentation cells (APCs), and as immunopotentiators, increasing immunogenicity without adverse reactogenicity [19, 20]. GVNPs are in the correct size range for eliciting cytotoxic T cell responses and have desirable surface charges and hydrophobic properties for interactions with APCs and phagocytes [21, 22].

A significant advantage of GVNPs as an adjuvant and antigen delivery system is their stability under a wide range of conditions and outstanding biocompatibility [18]. These nanoparticles maintain stability for extended periods of time even in the absence of a cold chain and have no known toxic effects in animals, either systemically or at the site of administration [23, 24]. GVNPs may be administered through conventional routes, by needle injection, subcutaneously or intraperitoneally (IP), as well as through alternate routes, including oral or nasal, and transdermally, using microneedles. These options may elicit both systemic and mucosal immunity, while simultaneously reducing costs and improving compliance.

Over the past 15 years, antigen display on GVNPs has been demonstrated using diverse peptide and protein antigens fused to the GvpC nanoparticle surface protein [18, 25–27]. The source of antigens displayed on the surface of GVNPs have thus far included a virus, simian immunodeficiency virus (SIV), two bacteria, the obligate intracellular pathogen *Chlamydia trachomatis* and the facultative intracellular pathogen *Salmonella enterica*, as well as the eukaryotic parasitic protozoan *P. falciparum* [23, 28–33]. GVNP-displayed antigenic proteins have ranged from secreted proteins to coat and envelope proteins, to transcription factors. Studies have been conducted both in vitro and in vivo, with displayed antigens being released slowly over days, and tested by challenge in the case of *Salmonella* [32, 33].

In the present study, a highly conserved 15-amino acid peptide sequence from the *Plasmodium* moonlighting protein enolase was cloned into the *Halobacterium* GVNP-display system [18]. The ability of this sequence displayed on GVNPs to elicit an antibody response that can protect against subsequent parasite challenge was tested in mice. The results showed that this approach indeed confers survival advantage to immunized mice against malaria.

## Methods

### Materials

Antibodies against rPfeno were raised in mice using in-house facilities as described earlier [34]. HRP-conjugated mouse and rabbit secondary antibodies were obtained from AbCam. ABTS [2,2′-azino-bis(3-ethylbenzothiazoline-6-sulphonicacid)] was obtained from Sigma-Aldrich Co.

### Purification of rPfeno and raising anti-rPfeno antibodies

GST-tagged-rPfeno was expressed in *Escherichia coli* and purified as described earlier [17]. Briefly, bacterial culture overexpressing rPfeno were homogenized and clear supernatant was obtained by centrifugation. GST-tagged recombinant protein was purified by passing the supernatant on a glutathione-Sepharose column. The protein was cleaved off its GST tag using thrombin. Purified rPfeno was recovered from the eluate. For raising antisera in mice, a published protocol was used as described earlier [34].

### Design, cloning and purification of gas vesicle nanoparticles

A double-stranded oligonucleotide was synthesized coding for a Pfeno derived 15-amino acid peptide (DGSKN**EWGWS**KSKLG, containing the *Plasmodium*^104^EWGWS^108^ epitope). The oligonucleaotide sequence was codon-optimized using the codon usage table for predicted genes in the fully sequenced *Halobacterium* sp. NRC-1 genome [35]. The synthetic DNA fragment was cloned into the *Halobacterium* sp. pSD104 vector at the *Afe*I site in *gvp*C [27], to construct plasmid pSD104::Eno. The *gvp*C gene region of the constructed plasmid was sequenced to verify the correctness of the insertion sequence.

Plasmid pSD104::Eno was transformed into *Halobacterium* strain SD109, a strain that lacks the gas vesicle gene cluster. After lysis of cells, Rec-GVNPs displaying the Pfeno epitope were purified from *Halobacterium* SD109 (pSD104::Eno) by flotation of the GVNP particles using the centrifugally accelerated procedure reported previously [36]. Presence of the GvpC-Pfeno fusion protein was confirmed by Western blotting using Pfeno primary antibody followed by alkaline phosphatase-conjugated goat anti-rabbit IgG secondary antibody (Sigma-Aldrich, St. Louis, MO, USA).

### Gel electrophoresis and Western blotting

SDS-PAGE was run using the Laemmli method [37]. Proteins were resolved on a 10 % SDS-PAGE and visualized by staining with Coomassie Brilliant Blue R-250. For Western blotting, proteins were separated by SDS-PAGE (10 % gels) and transferred to a PVDF membrane using semi-dry western transfer apparatus (Trans-blot SD-cell, Bio-Rad Laboratories, Inc., Hercules, CA, USA) at a constant voltage of 18 volts for 50 min. The membranes were blocked with 5 % skimmed milk in phosphate buffered saline containing 0.05 % Tween-20 for 2 h. The blots were treated with primary antibody. For the detection of *Plasmodium yoelii* (Pyeno) or Pfeno derived peptide sequence in Rec-GVNPs, anti-rPfeno antibodies were used at 1:1000 dilutions. Anti-rPfeno antibodies were raised in mice as described earlier [34]. Incubation with the primary antibody was followed by washing and incubation with HRP-conjugated secondary antibody. The immunoblots were developed using di-anilino-benzene substrate.

### Immunization of mice with nanoparticles

Experiments were performed on male Swiss mice aged 6–8 weeks. Mice were injected with 100 μg of WT-GVNPs or Rec-GVNPs in Freund’s complete adjuvant intra-peritoneally. Subsequent booster doses of 100 μg WT or Rec-GVNPs in Freund’s incomplete adjuvant were administered at 21 days interval. Ten days after the second booster sera from these animals were collected by partial retro-orbital bleed to assay for their reactivity against rPfeno, WT-GVNPs and Rec-GVNPs. All immunized mice and an un-immunized control group of mice were challenged with equal numbers of (approximately 10^6^) *P. yoelii* 17XL infected RBCs and their survival as well as percent parasitaemia was monitored as described earlier [15].

### ELISA

ELISAs were performed as previously described [34]. Briefly, the antigen in a volume of 100 μl was coated onto a Maxisorp immunoplate and incubated at 37 °C for 2 h. This was followed by washing the plate and blocking the wells with 5 % skimmed milk in PBST (10 mM Na_2_HPO_4_, 1.8 mM KH_2_PO_4_, 137 mM NaCl, 2.7 mM KCl containing 0.05 % Tween-20; pH 7.4) for 1 h at room temperature or 4 °C overnight. The blocking solution was discarded and the wells were washed with PBST three times for 5 min each. 100 μl of primary antibody (dilution varied according to experiment) was added to each well and incubated at room temperature for 1 h. The solution was discarded and the wells were washed with PBST three times to remove any unbound antibody. This was followed by the addition of HRP-conjugated anti-mouse secondary antibody at a dilution of 1:1000 and incubated at room temperature for 45 min. The solution was discarded and the wells were washed with PBST thrice. 200 μl of ready-to-use ABTS substrate was added into each well. The colour was allowed to develop for 10–15 min and absorbance was measured as an optical density (OD) at 405 nm on a Tecan Plate Reader.

For the measurements of anti-WT-GVNP (or anti-Rec-GVNP) antibody titres in serum, ELISA plates were coated with 1 µg of the respective antigen and sera were diluted 1:1000. For anti-rPfeno antibody titer determination, plates were coated with 100 ng of rPfeno and serum dilutions used were 1:100.

### Statistical analysis

For the comparison of survival data, Kaplan–Meier Log-Rank test was done. For all other analysis, Student’s *t* test was used (computed using Excel).

## Results

### Cloning and preparation of recombinant GVNPs from *Halobacterium* sp. NRC-1

In order to display the Pfeno antigenic epitope on GVNPs, an oligonucleotide coding for a Pfeno derived peptide containing the desired EWGWS sequence within an 15-amino acid sequence (DGSKN**EWGWS**KSKLG) was selected. This peptide sequence was BLASTed against the mouse genome (GenBank taxid:10088) to detect if there are homologous sequences present. However, no significant homology in mammalian genome was found. Use of a longer sequence that encompassed the conserved residues on either side of EWGWS ensured that the epitope was displayed in the proper Pfeno context. The oligonucleotide corresponding to the 15aa peptide was inserted at an *Afe*I site in *gvp*C in the *Halobacterium* sp. pSD104 vector, which contains the complete 14-gene gas vesicle gene cluster. The resulting plasmid pSD104::Eno was transformed into *Halobacterium* strain SD109, deleted for entire the gas vesicle gene cluster. GVNPs were purified from the resulting *Halobacterium* SD109 (pSD104::Eno) strain and wild-type strain NRC-1 by centrifugally accelerated flotation using a published procedure [27].

### Cloned sequence is recognized by anti-rPfeno antibodies

**Fig. 1:**
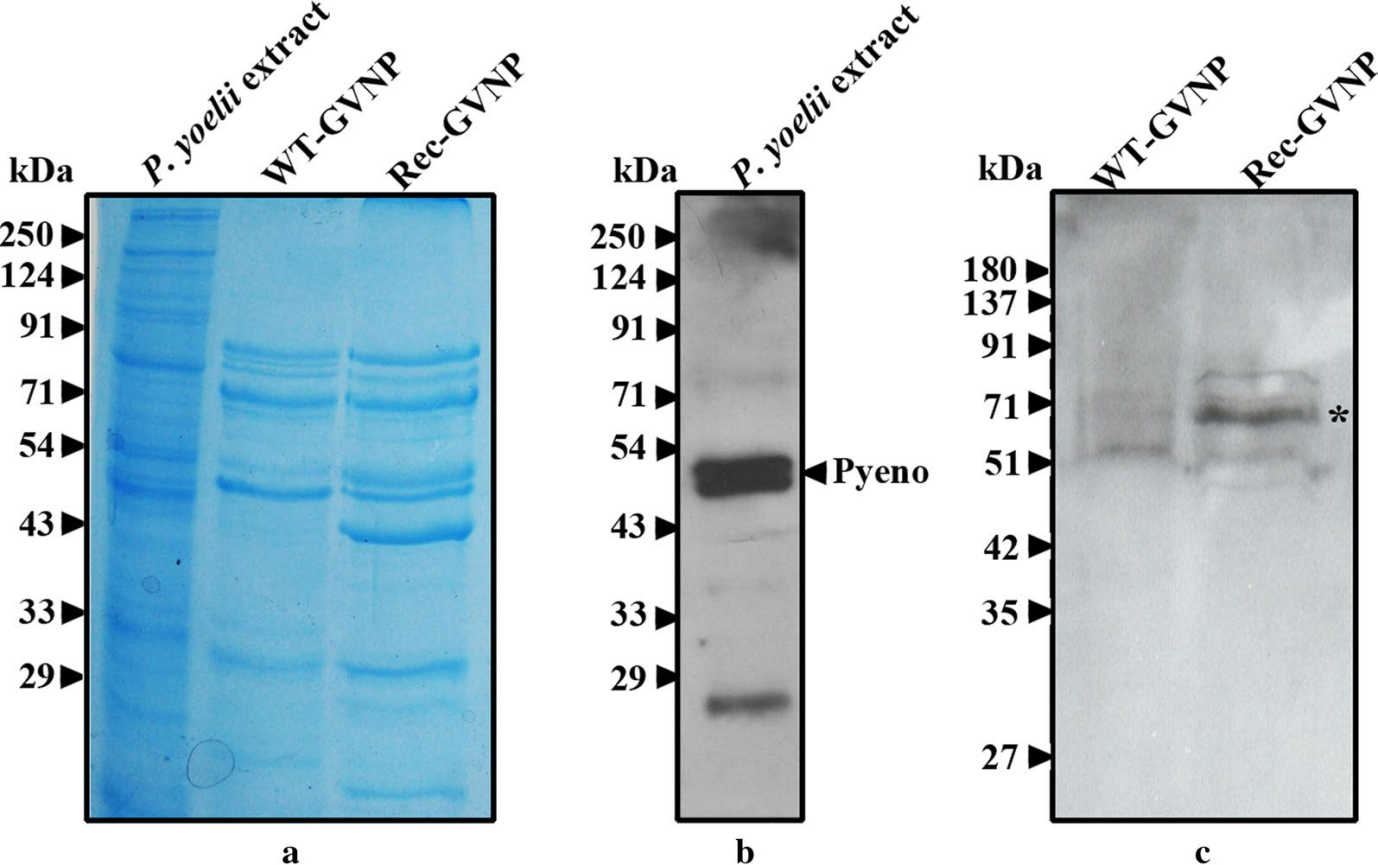
Protein profiles of the WT and Rec-GVNPs and their reactivity towards anti-rPfeno antibodies. **a** Coomassie stained 10 % SDS-PAGE gel showing protein profiles of *P. yoelii* cytosolic extract, WT-GVNPs and Rec-GVNPs. Western blots of **b** *P. yoelii* extract and **c** WT and Rec GVNPs using anti-rPfeno antibodies. Antibodies recognized Pyeno (~50 kDa) and a positive band observed at ~71 kDa (marked with *asterisk*) in Rec-GVNPs indicates the presence of parasite enolase derived peptide (EWGWS) insert

Wild-type nanoparticles (WT-GVNPs) differ from recombinant nanoparticles (Rec-GVNPs) in the presence of the Pfeno derived peptide ^104^EWGWS^108^ in the *gvp*C gene. For immuno-detection of this epitope in Rec-GVNPs and absence in WT-GVNPs for, samples of purified WT and Rec-GVNPs were analysed on a 10 % SDS-PAGE gel. *P. yoelii* cell extract (soluble fraction) was included as a positive control for antibody recognition of enolase. The protein profiles of WT and Rec-GVNPs are shown in Fig. 1a. Western blots with anti-rPfeno antibodies (mouse) are shown in Fig. 1b, c. Antibodies recognized enolase (MW ~50 kDa) in the parasite extract. Enolase positive bands at ~20 kDa and around 35–40 kDa were also observed in *P. falciparum* extracts and were identified by mass spectrometry to be the products of proteolysis of native 50 kDa form [38]. In the nanoparticle lanes, Western blot showed an enolase positive band with an apparent molecular mass of ~71 kDa (marked with *). This was present only in Rec-GVNPs and was absent in WT-GVNPs. The positive reactivity of the ~71 kDa band with anti-rPfeno polyclonal antibodies is due to the presence of the cloned sequence EWGWS in the GvpC-Eno protein found on the GVNPs and confirms the presence of the engineered peptide insert in the Rec-GVNPs.

### Immunogenicity and antibody specificity profile of nanoparticles

For comparative analysis of the antibody response, two groups of mice were immunized with WT-GVNPs (n = 5) and Rec-GVNPs (n = 10), respectively. A group of five mice (n = 5) were kept as un-immunized controls. For immunization, 100 µg of GVNPs in complete Freund’s adjuvant were injected. Subsequently, two booster doses (100 µg GVNPs in Freund’s incomplete adjuvant) were administered at an interval of 21 days. Serum samples were collected by partial retro-orbital bleed 10 days after second booster dose.

**Fig. 2:**
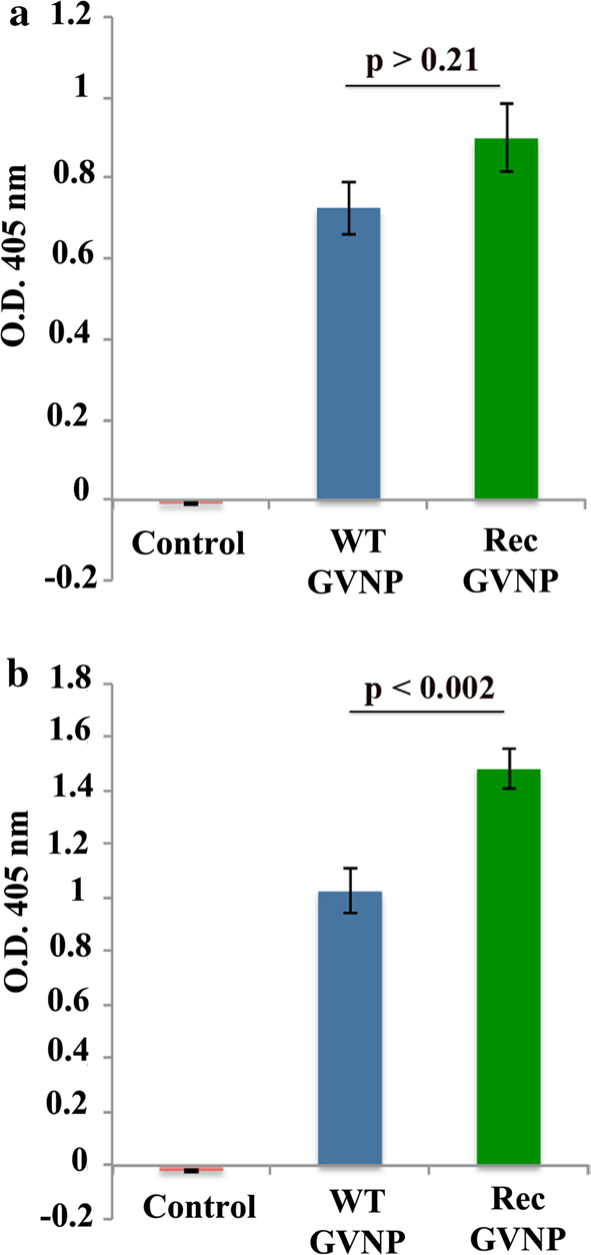
ELISA for the determination of antibody titres in serum samples derived from control (un-immunized), WT-GVNP and Rec-GVNP-immunized mice. Plates were coated with (**a**) WT-GVNPs and (**b**) Rec-GVNPs. The *error bars* represent standard deviation for the triplicates. Control (un-immunized) sera showed no reactivity. Reactivity towards WT-GVNPs was similar in the two groups (p > 0.21) (WT-GVNP and Rec-GVNP-immunized) **a**; however, reactivity towards Rec-GVNPs was significantly higher (p < 0.002) **b**. Number of mice in each group were—Control n = 5, WT-GVNP n = 5; Rec-GVNP n = 10. Seven mice with highest anti-rPfeno antibody titres in the Rec-GVNP immunized group were selected for the parasite challenge

Antibody responses to the two classes of particles (WT-GVNPs or Rec-GVNPs) were quantified by ELISA using one or the other particle as the antigen. ELISA measurements where the plates were coated with WT-GVNPs (1μg/well), both serum samples gave similar titres of antibodies (Fig. 2a) indicating that both groups of mice produced antibodies against the GVNP component proteins. However, when Rec-GVNPs were used as the antigen, higher antibody titres were observed with serum samples derived from Rec-GVNP-immunized mice (Fig. 2b). This was indicative of antibody response against the Pfeno derived peptide displayed on Rec-GVNPs. However, such a differential antibody response could also arise due to some contaminant in purified Rec-GVNPs. As expected, serum samples from un-immunized control mice did not show any reactivity towards either of the particles when used as antigens (Fig. 2a, b). Lack of differential reactivity towards WT-GVNPs between the two types of serum samples (raised against WT-GVNPs and Rec-GVNPs) suggested that coated WT-GVNPs reacted with the antibodies arising against similar epitopes in both samples. However, the higher reactivity towards Rec-GVNPs in serum samples from Rec-GVNP immunized mice suggested that a fraction of antibodies was directed against the cloned sequence DGSKN**EWGWS**KSKLG containing EWGWS, the parasite specific insert motif of Pfeno. To determine whether this is indeed the case, the reactivity of sera using rPfeno as an antigen was measured.

### Immunization with recombinant nanoparticles generate antibody responses specific to rPfeno

**Fig. 3:**
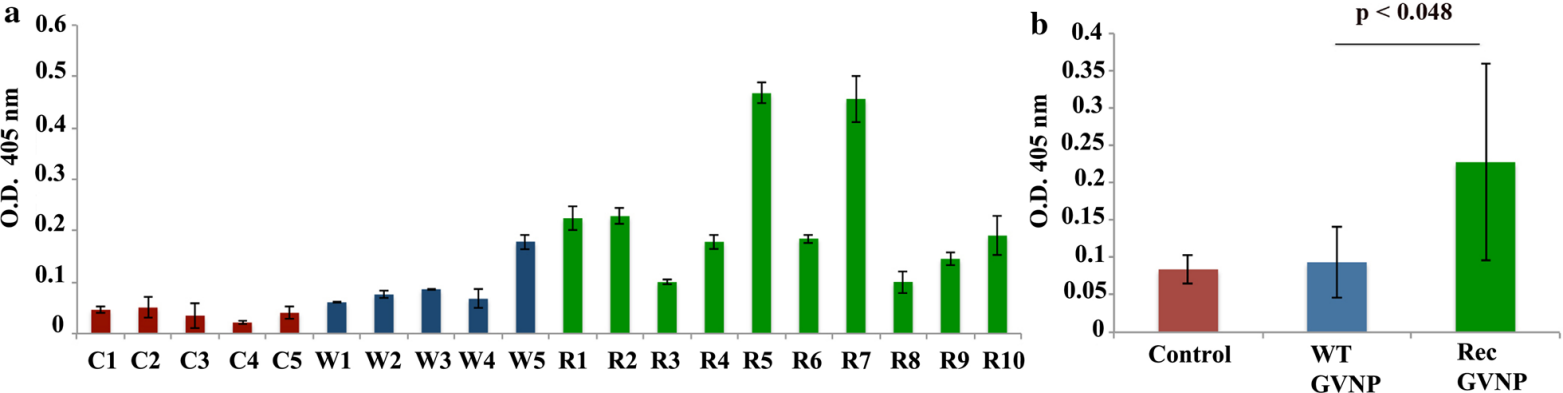
Anti-rPfeno antibody titres in serum samples derived from un-immunized (C1-C5), WT-GVNPs (W1-W5) and Rec-GVNP (R1-R10) immunized mice. **a** ELISA for the measurement of anti-rPfeno antibody titres in serum obtained from individual mice. **b** *Bar graph* showing average anti-rPfeno antibody titres. Antibody response was significantly higher in Rec-GVNP-immunized mice as compared to WT-GVNP-immunized ones (p < 0.048)

To assess the immunogenic potential of *Halobacterium* gas vesicle nanoparticles displaying the Pfeno derived peptide in the mouse system, sera from WT and Rec-GVNP-immunized mice were subjected to ELISA using rPfeno as an antigen. 100 ng of rPfeno was coated onto each well of the plate and 1:100 diluted sera from immunized and control mice was used as primary antibody. Results of the experiment for serum reactivity of each individual mouse are shown in Fig. 3a. Figure 3b shows the average values for all the animals in a single treatment group. Anti-rPfeno antibody titres were found to be significantly higher in the case of Rec-GVNP-immunized mice as compared to the WT-GVNP group (Fig. 3b). Although, it was expected that displayed Pfeno peptide in the context of gas vesicle nanoparticles (Rec-GVNPs) would elicit a robust antibody response, observations indicate the antibody response was relatively mild which is not unexpected, given the proportionate size of the 5 amino acid sequence compared to the entire GVNP complex.

### Protective effect of immunization with Pfeno peptide

**Fig. 4:**
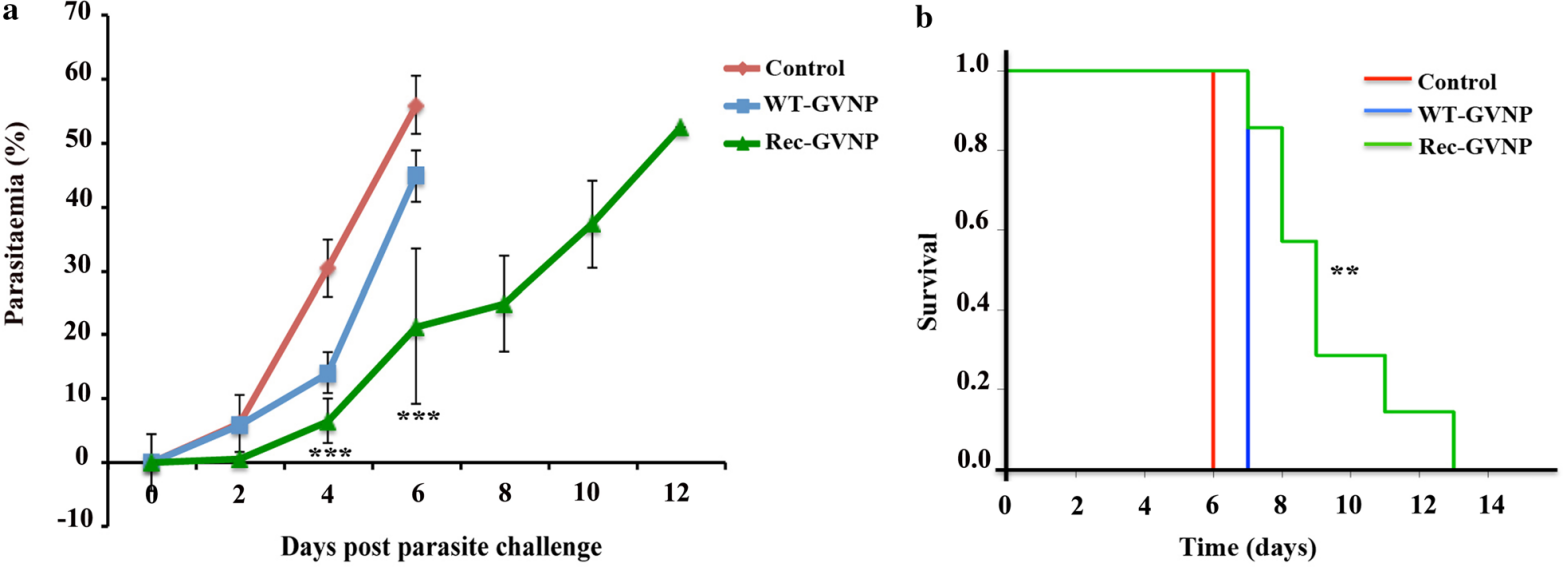
Rec-GVNP-immunized mice showed low parasitaemia and longer survival as compared to un-immunized and WT-GVNP-immunized groups. All three groups of mice were injected intra-peritoneally with equal number of *P. yoelii* 17XL iRBCs (approximately 10^6^). **a** % Parasitaemia significantly differed on days 4 and 6 for the Rec-GVNP immunized mice (p = 0.001); **b** survival profile of mice as a function of days

To evaluate whether the antibodies against the parasite specific insert sequence of Pfeno are protective under in vivo conditions, all three groups of mice, viz. un-immunized (control), WT and Rec-GVNP-immunized mice, were challenged with equal numbers of iRBCs (approximately 10^6^) infected with asynchronous *P. yoelii* 17XL (lethal strain), which were isolated from infected mice. The parasites were injected 10 days after the second booster dose of nanoparticles. Parasitaemia in these mice was monitored on every second day post parasite injection to assess the effect of immunization on parasite growth. Percent parasitaemia in the control groups of mice (i.e. un-immunized and WT-GVNP-immunized) was much higher as compared to Rec-GVNP-immunized group. Statistical analysis of data using Student’s t-test showed that on days 4 and 6 (post parasite challenge), WT-GVNP-immunized group had significantly higher percent parasitaemia (p = 0.001) as compared to Rec-GVNP-immunized group. The extent of parasite growth retardation in Rec-GVNP immunized mice was approximately 2- to 2.5-fold as compared to the ones immunized with WT-GVNPs (Fig. 4a).

These mice were also monitored for their survival. Survival profiles for all three groups are shown in Fig. 4b. Rec-GVNP-injected mice survived significantly longer compared to WT-GVNP-treated and un-immunized control groups. As stated above, although the antibody titres of anti-EWGWS in Rec-GVNP immunized mice were moderate, observation of significant retardation in parasite growth and prolongation of survival support the view that the Pfeno derived peptide constitutes a protective antigenic epitope against malaria.

### Percent parasitaemia and survival profile correlates with anti-rPfeno antibody titres in Rec-GVNP immunized mice

**Fig. 5:**
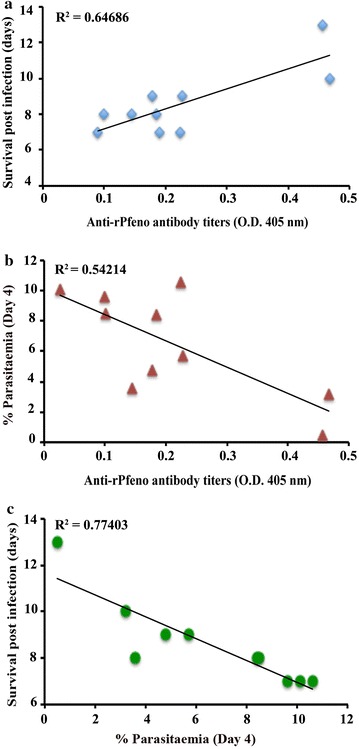
Correlation between anti-rPfeno antibody titres in Rec-GVNP-immunized mice with **a** survival (post infection days), **b** % parasitaemia (on day 4) and **c** % parasitaemia vs survival. Linear fit of data gave **a** a positive correlation (R^2^ = 0.64) between survival and antibody titres; **b** a negative correlation (R^2^ = 0.54) for the parasite growth and **c** a negative correlation (R^2^ = 0.77) between % parasitaemia and survival

Since each Rec-GVNP-injected mouse had a different degree of reactivity with rPfeno on the ELISA based assay (Fig. 3a), it indicated that the antibody titres against rPfeno in each immunized animal was different. If EWGWS was the protective epitope, titres of antibodies against it should have a positive correlation with survival and negative with percent parasitaemia. Anti-rPfeno antibody titres as measured by ELISA were plotted against the number of post parasite challenge days that an animal survived (Fig. 5a). The plot showed a positive correlation (R^2^ = 0.64) (albeit weak) between survival and antibody titres. Antibody titre vs. percent parasitaemia was also plotted. It was expected that anti-EWGWS antibodies would inhibit parasite growth if this sequence constitutes a protective epitope. The plot did show a negative correlation (Fig. 5b), i.e. the increase in antibody titres interfered with the growth and division of the parasite resulting in a negative correlation between percent parasitaemia and anti-rPfeno antibody titres. A negative correlation between percent parasitaemia and number of days a mouse survived post-challenge of the lethal parasite was also observed (Fig. 5c), indicating that the parasite load is a critical factor in the survival of the mice. These results suggested that anti-EWGWS antibodies inhibited the growth of the parasite, resulting in diminished parasitaemia, and that in turn brought about enhanced survival.

## Discussion

Development of an effective vaccine and delivery system for malaria is urgent. While a large number (~100) of candidate malaria vaccines are being tested, most are based on only a handful of antigens and standard modes of immunization [3, 39]. Given the prevalence of the disease, there is a compelling need for identifying additional antigens with protective properties and testing novel display and delivery systems. One such antigen candidate is enolase which has been shown to possess invasion functions when present on the surfaces of *Plasmodium* spp. merozoites and ookinetes [11, 14, 15]. The potential of a short peptide sequence (EWGWS) derived from the parasite was displayed on *Halobacterium* gas vesicle nanoparticles for studying its protective antigenic properties. The results obtained are encouraging and warrant further studies, especially since the high stability of GVNPs provides the potential for distribution of a vaccine in the absence of a cold-chain [18].

For an effective vaccine, it is essential that the antigen induces a long lasting robust immune response (resulting in secretion of antibodies and generation of memory B-cells) and activation of CD8+ T-cells that constitutes a significant challenge. Use of a combination of multiple antigens in conjunction with different adjuvants have not been very successful in obtaining strong B and T cell responses [3]. Effective induction of immune activation was observed in several recent studies where nanoparticle based vaccines were used [40–42]. The strategy used in this study, was to insert a *Plasmodium*-specific antigenic peptide sequence in a protein that is a component of the GVNPs shown to be released slowly in the immune system [29, 30]. Presence and display of this sequence in Rec-GVNP was verified by the observed reactivity of anti-rPfeno antibodies in Western blots (Fig. 1c) as well as in ELISAs (Fig. 3).

Mice that were immunized with Rec-GVNPs having the Pfeno epitope, when challenged with *P. yoelii* 17XL (~10^6^ iRBCs per animal), showed significantly slower growth of parasitaemia as compared to the control and WT-GVNP-immunized cohorts (Fig. 4a). Inhibition of parasite growth is likely to be due to the antibody response directed against the cloned parasite enolase sequence in the Rec-GVNP-immunized mice. Further, monitoring these mice for survival showed significant prolongation of survival as compared to other groups. Correlation between anti-rPfeno antibody titres and parasitaemia as well as survival for individual mice was also analysed. As shown in Fig. 5a, b, a positive correlation was found with survival, i.e. higher levels of anti-rPfeno antibodies led to longer survival of animals. Conversely, a negative correlation was observed where higher antibody titres led to reduction in parasitaemia. As expected, a mouse with higher parasitaemia died earlier (Fig. 5c). These results strongly support the conclusion that the Pfeno sequence EWGWS is a protective epitope.

Functionally, the -EWGWS- motif is likely to be involved in interaction with the cell surface receptors in RBCs and mosquito midgut epithelial cells. Recent studies on the functional role of the motif in the biochemical activity of the enzyme suggested that it could stabilize the apo-enzyme in an active form [43]. Although immunization with Pfeno derived peptide conferred longer survival and controlled the growth of parasitaemia, the overall effect was rather modest. This was also evident from the observed titres of antibodies against rPfeno in Rec-GVNP-immunized mice (Fig. 3a, b). and likely reflects the small size of the antigenic epitope.

In recent studies, the results with *Halobacterium* GVNPs have been encouraging for the display of *Salmonella*, *Chlamydia*, SIV antigens, as well as *Plasmodium* CSP [18, 23, 28–33]. Although the parasite specific unique insert EWGWS in Pfeno is likely to be a protective antigenic epitope, future efforts should be directed at engineering nanoparticle preparations that elicit stronger antibody responses in order to achieve a complete clearance of the parasite. A promising approach in this regard may be to display multiple antigens that could lead to a more protective formulation [27, 44].

It has been noted that people in endemic areas have the potential to develop protective immunity following multiple episodes of malaria. IgGs isolated from such people inhibit parasite growth in vitro [45] and passive transfer to malaria patients can lead to recovery [46]. Consequently, identification of antigens that are recognized by serum antibodies derived from immune individuals but not by malaria patients has been one approach to identifying vaccine candidates. Using such differential screens, new antigens like PfP0 [47] and PfSEA-1 [48] with protective antigenic properties have been identified and offer further opportunities for application using the GVNP technology.

## Conclusions

Data presented here demonstrate that a parasite-specific peptide sequence derived from Pfeno and displayed on GVNPs is able to elicit an antibody response against enolase when injected in mice. Anti-peptide antibodies elicited protective responses as evident from low parasitaemia and enhanced survival of Rec-GVNP-immunized mice. These experiments support the existence of a protective antigenic epitope in parasite enolase and our ability to display and deliver this sequence on GVNPs. Further efforts are needed to enhance the immunogenicity and protective potential of this potential malaria vaccine candidate.

## Abbreviations

rPfeno: recombinant *Plasmodium falciparum* enolase
Pyeno: *Plasmodium yoelii* enolase
WT-GVNPs: wild type gas vesicle nanoparticles
Rec-GVNPs: recombinant gas vesicle nanoparticles
RBCs: red blood cells
iRBCs: infected RBCs
